# Elevated Rates of Sister Chromatid Exchange at Chromosome Ends

**DOI:** 10.1371/journal.pgen.0030032

**Published:** 2007-02-23

**Authors:** M. Katharine Rudd, Cynthia Friedman, Sean S Parghi, Elena V Linardopoulou, Li Hsu, Barbara J Trask

**Affiliations:** 1 Division of Human Biology, Fred Hutchinson Cancer Research Center, Seattle, Washington, United States of America; 2 Division of Public Health Sciences, Fred Hutchinson Cancer Research Center, Seattle, Washington, United States of America; The Jackson Laboratory, United States of America

## Abstract

Chromosome ends are known hotspots of meiotic recombination and double-strand breaks. We monitored mitotic sister chromatid exchange (SCE) in telomeres and subtelomeres and found that 17% of all SCE occurs in the terminal 0.1% of the chromosome. Telomeres and subtelomeres are significantly enriched for SCEs, exhibiting rates of SCE per basepair that are at least 1,600 and 160 times greater, respectively, than elsewhere in the genome.

## Author Summary

The ends of chromosomes are evolutionarily dynamic and structurally unusual parts of the human genome. Arrays of telomeric repeats cap each end and protect chromosomes from degradation and end-to-end fusions. Just inside the telomeres are patchworks of larger DNA segments duplicated on different chromosome ends. These subtelomeric duplications reflect the high frequency with which DNA breaks in these regions were healed by interchromosomal repair processes during recent primate evolution. In this study, we asked if chromosomal ends are also unusually susceptible to replication-induced DNA breaks and repair during mitotic division of somatic cells. We employed a specialized fluorescent technique to measure sister chromatid exchange (SCE) specifically in telomeres and subtelomeres, as such events would be missed by standard SCE-detection methods. We find extraordinarily high rates of SCE in these terminal regions: over 15% of observed SCEs occur in just 0.1% of the genome. Thus, chromosome ends are hotspots of DNA breaks and recombinational repair in mitosis, as shown previously in meiosis. The enrichment of DNA breaks at chromosome ends contributes to normal variation, chromosome evolution, and chromosome rearrangements leading to disease.

## Introduction

**Figure 1:**
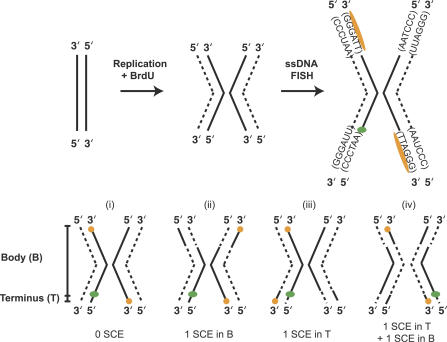
CO-FISH Methodology Cells were synchronized at the G1/S boundary and allowed to replicate in the presence of BrdU. Replicated chromosomes have chromatids with one BrdU-incorporated DNA strand (dashed line) and one original DNA strand (solid line). The BrdU-incorporated strand is digested so that it is unavailable for hybridization. The single-stranded telomere probe (orange) and one single-stranded internal subtelomeric probe (green) hybridize to the non-BrdU-labeled strand. Each of four internal probes was cohybridized with the telomeric probe in a separate experiment. Telomere repeats, (TTAGGG)_n_, are oriented 5′ to 3′, so the orange telomeric PNA probe, (CCCTAA)_3_, hybridizes to one chromatid on each chromosome end. Fully degraded and hybridized chromosomes may exhibit one of four probe configurations. (i) If no SCE has occurred during or subsequent to replication, telomeres are oriented in a diagonal *(trans)* pattern, and the internal probe, because of its 5′–3′ orientation, hybridizes to the chromatid opposite the nearest telomere signal. (ii) If one SCE, or an odd number of SCEs, occur(s) in the body of the chromosome (B), as indicated by switched dashed and solid lines, the telomere probes hybridize to both ends of the same chromatid (in a *cis* pattern), but the internal probe remains at the same position relative to the closest telomere signal (i.e., on the opposite chromatid). (iii) If one or an odd number of SCEs occurs in the terminal interval (T) between the subtelomeric internal probe and the telomere, all probe signals will lie on the same chromatid. (iv) If one SCE occurs in the body and one occurs in the terminal interval of the chromosome, the telomere probe signals will appear in their original diagonal *trans* configuration, but the internal subtelomere probe will shift to the opposite chromatid than in (i) and end up on the same chromatid as the nearby telomere signal.

Chromosome ends participate in frequent recombination events. Human subtelomeric regions have undergone multiple interchromosomal exchanges during meiosis, giving rise to the highly duplicated structures proximal to telomere repeats [1]. Meiotic recombination maps of the human genome show an increase in recombination rate at the most distal markers, especially in males [2,3]. Other observations suggest that mitotic recombination might also be elevated at chromosome ends. In senescent cells, telomeres and subtelomeres are enriched for double-strand-break–binding proteins such as γ-H2AX [4]. Telomeric recombination is elevated in telomerase-negative cancer cells that follow the alternative lengthening of telomeres (ALT) pathway, generating chromosomes with highly variable telomere lengths [5–7]. Based on these somatic observations and the evolutionary dynamics of human subtelomeres, we hypothesized that subtelomeres might undergo high rates of mitotic sister chromatid exchange. Sister chromatid exchange (SCE) is a mechanism that resolves replication-dependent double-strand breaks and is thus an indicator of DNA damage and repair. Using a novel fluorescent method to detect SCEs anywhere between two chromosome ends (chromosome orientation fluorescence in situ hybridization [CO-FISH] [8]), Cornforth and Eberle (2001) observed more SCEs than found using classical harlequin SCE techniques [9]. They attributed this excess to the most distal ≤7 Mb of the chromosomes. We have adapted CO-FISH to specifically measure SCEs in the telomeres, subtelomeres, and body of chromosomes (Figure 1) and find that SCEs are highly concentrated within the most distal 100 kb.

## Results

**Figure 2:**
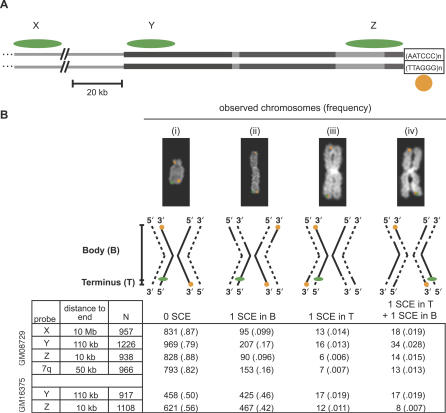
CO-FISH Probe Map and Frequency of SCE (A) Single-stranded CO-FISH probes X, Y, and Z, shown as green ovals, hybridize 10 Mb, 110 kb, and 10 kb from the end of chromosomes, respectively. Y and Z probes hybridize to duplicated subtelomeric sequences, shown as grey rectangles. The orange probe indicated by the circle hybridizes to the telomere-repeat sequence (TTAGGG)_n_ at the ends of all chromosomes. The amount of SCE occurring between the orange telomeric signal and each green probe signal was measured in separate experiments. The scale bar indicates 20 kb. (B) In each experiment, four different CO-FISH configurations are possible in fully processed and hybridized chromosomes, shown as examples and diagrams as in Figure 1. The number of chromosomes (and relative frequency) observed in each configuration is shown below. SCE events within the telomere that split the telomere probe signal between two chromatids at the same end were counted separately and not included in our estimates of terminal SCE rates (Text S1). GM08729 is a normal lymphoblastoid cell line, and GM16375 is a lymphoblastoid cell line derived from a patient with Bloom syndrome.

We measured SCE in three terminal intervals of the chromosome, the distal 10 Mb (probe X), the subtelomere (the terminal ~110 kb, probe Y), and the most distal ~ 10 kb including the telomere (probe Z). Three separate experiments were conducted by cohybridizing a probe specific for the telomere-repeat sequence, (TTAGGG)_n_, with one of the three internal probes (X, Y, or Z) (Figure 2). A change in the chromatid position of the internal probe relative to the telomere-repeat probe indicates an SCE in the interval between the signals of the two probes (Figure 1).

For example, Probe Z lies in the most distal part of the subtelomere just proximal to the telomere-repeat array, so it monitors SCE events that occur in the <10-kb interval between its green signal and the orange signal produced by the telomere-repeat probe at the same chromosomal end. In the native state, these signals reside on opposite chromatids, due to the opposing 3′–5′ orientations of the two probes at this chromosomal end. If an SCE occurs between the target sequences such that the bulk of the telomere-probe signal is transferred to the other chromatid, the signals of both probe Z and the telomere probe will lie on the same chromatid. Probe Z also monitors SCE events that occur between it and the telomere-probe signal at the far end of the chromosome; we refer to this interval as the body of the chromosome. These events are recognized by the shift from the native configuration (i in Figure 1) to the configuration diagrammed in ii, Figure 1. SCEs that occur precisely within the sequences targeted by either probe will go undetected unless sufficient sequence is transferred to the opposite chromatid to be made visible by FISH. For example, since the telomeric arrays of human cells are relatively short (<10 kb), many SCEs that occur within the telomere proper are missed because too little telomeric sequence is transferred (or left behind) to produce signals on both chromatids at the same chromosome end. We rarely detected such double telomere-probe signals in normal (GM08729) cells (0.4% of labeled chromosome ends on average, Text S1). These double signals could represent telomeric SCEs, but since they are relatively rare in normal cells and might also arise artifactually due to incomplete degradation of the newly synthesized DNA strand, we chose to omit this small group of chromosomes from our estimates of terminal SCEs to be conservative.

Our telomeric and subtelomeric probes each hybridize to multiple chromosomes at approximately the same distance from the ends, whereas the probe assaying the distal 10 Mb hybridizes only to the long arm of Chromosome 15 (Figure 2 and Table S1). We counted the number of SCE events in the body of the chromosome and in each of the three terminal intervals in a lymphoblastoid cell line. Pooling SCE data from all chromosomes with the requisite signals (internal probe + telomere probes on both p and q ends), we find that the frequency of chromosomes with a single terminal SCE in the most telomeric interval (Z), subtelomere (Y), and distal 10 Mb (X) is 0.6%, 1.3%, and 1.4%, respectively. Thus, the majority of SCEs in the last 10 Mb of a chromosome are confined to the distal 110 kb. Of all the SCE events we observed, 17% (50/291) occurred in the last 110 kb and 16% (20/124) occurred in the last 10 kb of the chromosome, far greater proportions than would be expected if SCEs were distributed uniformly along the chromosome. The frequency of observed SCEs in the terminal intervals implies rates of 2,100 × 10^−9^ SCE/(bp × cell generation), 380 × 10^−9^ SCE/(bp × cell generation), and 3.3 × 10^−9^ SCE/(bp × cell generation) in the terminal 10 kb, 110 kb and 10 Mb, respectively (Text S1; Table S2).

Recent studies in budding yeast have shown that subtelomeric sequences direct nuclear organization [10], and that alterations in nuclear organization can affect the frequency of double-strand-break repair at subtelomeres [11]. Thus, we speculated that different human subtelomeres might have variable rates of SCE. However, our analysis of 16 different subtelomeres did not detect a chromosome-specific difference in terminal SCE rates (Table S1). The chromosome ends we sampled have undergone multiple interchromosomal exchanges during evolution giving rise to a patchwork of subtelomeric duplications. Therefore, we also measured SCEs in 7q, a chromosome end relatively devoid of duplications [1]. The rate of SCE in the last 50 kb of 7q is 420 × 10^−9^ SCE/(bp × cell generation), not significantly different from the rate of SCE on chromosome ends with more extensive interchromosomally duplicated content.

The frequency of chromosomes with an SCE in the body of the chromosome outside of the terminal interval varies among the four experiments due to the differences in the DNA contents of the analyzed chromosomes (Figure 2 and Table S1). On average, the rate of SCE in the body of the chromosome is 1.3 × 10^−9^ SCE/(bp × cell generation) (Text S1). Note that this rate for the body of the chromosome is inflated because it encompasses events occurring in the terminal interval of one chromosome end, since our internal probes usually only mark a single end. As in previous studies [9,12], our measurements of SCEs in 15 different chromosomes show that the frequency of total SCEs increases linearly with chromosome size (Figure S1).

Interestingly, in each of the four experiments, we found a significant number of chromosomes with two SCEs—one in the terminal interval and one in the remainder of the chromosome (Figure 2)—over what would be expected if SCEs were independent events (p < 0.0001; Text S1, 2 × 2 contingency table). We also found an excess of cells with more SCEs than expected from the overall average SCE rates (unpublished data). Thus, SCEs appear to cluster.

We also measured terminal SCE in a SCE-sensitized background. Cells from patients with a mutation in the gene encoding the Bloom syndrome protein, BLM, have significantly more SCEs along the length of the chromosomes than do normal cells [13]. BLM physically interacts with the telomere-binding protein TRF2 in HeLa cells and unwinds telomere duplexes in vitro [14]. Thus, we considered the possibility that BLM could play a role in the hyper-recombination occurring at chromosome ends. We measured SCE in the two most terminal intervals of chromosomes in cells from a patient with Bloom syndrome (Figure 2). The very high rates of SCE in the body of chromosomes in these cells saturates the CO-FISH assay; chromosomes with an even number of SCEs in the body of the chromosome will have the same probe configuration as the native state, whereas chromosomes with any odd number of SCEs in the body will be indistinguishable from chromosomes with one SCE in the body. In the Bloom cells, the frequencies of the two configurations are equivalent, due to the multiplicity of events they represent. In contrast, the frequency of chromosomes showing terminal SCEs in Bloom cells was very similar to the frequency observed in normal cells (Figure 2). Thus, cells lacking functional BLM do not have a proportional increase in terminal SCE.

Increased rates of exchange within the telomere-repeat array have been found in cancer cells following the ALT pathway [7,15,16]. ALT cells show an abundance of double telomere signals in CO-FISH assays, suggesting an increase in telomeric SCE and/or interchromosomal exchanges between telomere-repeat arrays on different chromosomes. To determine if subtelomeres were also involved in the terminal exchanges in ALT cells, we applied our subtelomeric CO-FISH assay to an ALT cell line (WI38 VA13/2RA). As in previous studies [7], we found an increase in double telomeric-probe signals in ALT chromosomes (18% versus 0.4% in normal), but we did not find double internal probe signals (probe Y) indicative of subtelomeric interchromosomal exchanges. The lack of subtelomeric interchromosomal exchanges in ALT cells is consistent with experiments using a non-native subtelomere [17]. Also, rates of SCE in the terminal 110 kb, exclusive of the telomere array itself, were not elevated in ALT cells relative to normal cells (unpublished data), although these SCEs could be detected only in the chromosomes without double telomere signals at the end carrying the subtelomeric probe. These data suggest that the ALT pathway uses telomeres and not subtelomeres as a substrate for chromosome-end maintenance.

## Discussion

Chromosome ends are an extremely dynamic part of the genome. CO-FISH allows us to measure SCE anywhere along the entire length of the chromosome, from telomere to telomere. We find that SCE is highly concentrated at the very ends of chromosomes, as over 15% of all mitotic SCEs occur in a region roughly 0.1% of the chromosome's length. The most distal ~10-kb regions show the greatest density of SCEs, at 2,100 × 10^−9^ SCE/(bp × cell generation). When we subtract these most telomeric SCEs from the number of SCEs in the last 110 kb of the chromosome, we find a rate of 210 × 10^−9^ SCE/(bp × cell generation) in the subtelomere alone. Both regions exhibit rates of SCE much greater than the rate elsewhere in the genome, approximately 1.3 × 10^−9^ SCE/(bp × cell generation). Our comparison of SCE rates is conservative, as the latter rate calculation does not correct for the excess of SCEs at the unanalyzed chromosome end. We do not find a significant difference in the frequency of SCEs at different chromosome ends, suggesting that terminal location alone may be sufficient for increased SCE.

The 160-fold and at least 1,600-fold enrichment of SCE in subtelomeres and telomeres, respectively, suggests that chromosome ends are subject to more double-strand breaks during replication and/or that they are more likely to be repaired by SCE than more internal regions of chromosomes. These data indicate that human subtelomeres are hotbeds of DNA repair and exchange during mitosis and complement earlier findings of high rates of recent meiotic exchange at chromosome ends [1,18]. While most exchanges between sister chromatids probably leave their DNA sequences unaltered, SCE is known to mediate somatic changes in the length of D4Z4-repeat arrays in the 4q subtelomere; severe shortening of this array causes facio-scapulo-humeral dystrophy [19,20]. Thus, subtelomeric SCE is a common event that can have pathologic consequences.

## Materials and Methods

### Cell lines.

Human lymphoblastoid cell lines GM08729 and GM16375 were obtained from the Coriell Institute for Medical Research (http://www.coriell.org) and grown in RPMI media supplemented with 10% fetal bovine serum, penicillin/streptomycin, and L-glutamine. SV40-transformed human fibroblast cell line WI38 VA13/2RA was obtained from the American Type Culture Collection (http://www.atcc.org) and grown in alpha-MEM supplemented with 10% fetal bovine serum, penicillin/streptomycin, and L-glutamine. Cells were harvested and prepared for CO-FISH as described by Cornforth and Eberle (2001). Figure 1 diagrams the CO-FISH procedure. Briefly, cells were synchronized at the G1/S boundary by serum starvation, released into S phase, and treated with 30 μM BrdU, allowing the newly replicated DNA strands to incorporate BrdU. Mitotic cells were harvested and dropped on slides as described [21]. After 1–7 d of storage at room temperature, slides were treated with 0.5 mg/ml RNase A for 10 min at 37 ^°^C, followed by rinsing in PBS. Next, slides were treated with 0.5 μg/ml Hoechst 33258 (Sigma) for 15 min and then exposed to 365-nm UV light for an additional 30 min. We used a TL-33E transilluminator (UVP, Incorporated; http://www.uvp.com), which operates at an intensity of 91,000 Joules/m^2^. Slides were washed in PBS and then chromosomes were digested with 100 μl of 3 U/μl Exo III (Fermentas, http://www.fermentas.com) for 5 min at room temperature. UV exposure followed by exonuclease digestion degrades BrdU-incorporated DNA strands, generating single-stranded chromatids. Finally, slides were ethanol dehydrated at room temperature by successively incubating them in 70%, 80%, 95%, and 100% ethanol for 2 min at each concentration, and allowed to air dry. Slides were denatured and hybridized to CO-FISH probes as described below.

### CO-FISH probes.

Single-stranded probes were constructed from four different genomic locations (X, Y, Z, and 7q). Each CO-FISH probe is a pool of several single-stranded plasmids with inserts cloned from a particular region of the genome. Probe X is composed of eight plasmids, cloned from bacterial artificial chromosome (BAC) RP11-24J19, which span approximately 50 kb of sequence about 10 Mb from the end of the long arm of Chromosome 15 (March 2006 University of California Santa Cruz (UCSC) browser [http://www.genome.ucsc.edu] coordinates chr15:90227457–90279975). It is not duplicated on other chromosomes. Probe Y is composed of four plasmids, cloned from P1-derived artificial chromosome RP5-855D21, which span approximately 20 kb of duplicated sequence located about 110 kb from the end of the short arm of Chromosome 8 (March 2006 UCSC browser coordinates chr8:102784–121678). Probe Z is located at the most distal region of many chromosomes, just proximal of telomere repeats [1]. The five plasmids in probe Z were cloned from BAC RP11-395L14 and span approximately 25 kb of sequence (March 2006 UCSC browser coordinates chr2:114050604–114075702). This BAC originates from the ancestral site of the telomere-telomere fusion on Chromosome 2, which contains sequences paralogous to subtelomeric sequences. The sequences of probes Y and Z are known from FISH and genome-sequence analyses to be duplicated on at least nine and thirteen chromosome ends, respectively, although the number and location varies among individuals ([1], and unpublished data). The eight plasmids comprising the 7q probe lie about 50 kb from the terminus of the long arm of Chromosome 7. Cloned from BAC RP11-1112M14, the 7q probe set spans approximately 78 kb (March 2006 UCSC browser coordinates chr7:158701668–158780077).

Each plasmid insert was TA-cloned from a PCR product in F′ E. coli (TOPO TA cloning kit; Invitrogen, http://www.invitrogen.com). Single-stranded DNA was generated by infecting F′ cultures with M13K07 helper phage according to the manufacturer's instructions (New England Biolabs, http://www.neb.com). 1 μg of single-stranded DNA was digested with 5 U of DNase I for 1 min at room temperature (New England Biolabs) and then end-labeled with biotin-16-ddUTP according to manufacturer's instructions (Roche Applied Science, http://www.roche.com).

Slides were treated to generate single-stranded chromatids, as described in [9] and outlined above. CO-FISH probes were denatured, hybridized to slides, and detected with avidin-FITC as described [21]. Following biotin detection, slides were hybridized with 10 μl of a 0.5 μg/ml telomere peptide nucleic acid probe (Cy3-[C_3_TA_2_]_3_) and washed as described [22]. Slides were covered with 20 μl of antifade solution with DAPI (Vectashield; Vector Laboratories, http://www.vectorlabs.com), allowing for identification of the chromosomes from their banding patterns. Signals were examined using a Zeiss fluorescence microscope (http://www.zeiss.com) equipped with a cooled CCD camera, Chroma Technology spectral filters (http://www.chroma.com), and MacProbe image analysis software (Applied Imaging Corporation, http://www.aicorp.com).

## Supporting Information

Figure S1 Correlation of Chromosome Size with SCE Frequency (1.5 MB DOC)

Table S1 CO-FISH Data Expressed per Chromosome (81 KB DOC)

Table S2 SCE Frequency Corrected for Double Exchanges (24 KB DOC)

Text S1 Methods and Calculations (64 KB DOC)

### Accession Numbers

The National Center for Biotechnology Information (NCBI) (http://www.ncbi.nlm.nih.gov) accession numbers for the genomic information discussed in this paper are BAC RP11-24J19, AC104236; PAC RP5-855D21, AC004908; BAC RP11-395L14, AL078621; and BAC RP11-1112M14, AQ747375 and AQ747941.

## Abbreviations

ALT: alternative lengthening of telomeres
BAC: bacterial artificial chromosome
CO-FISH: chromosome orientation fluorescence in situ hybridization
SCE: sister chromatid exchange

## References

[pgen-0030032-b001] Linardopoulou EV, Williams EM, Fan Y, Friedman C, Young JM (2005). Human subtelomeres are hot spots of interchromosomal recombination and segmental duplication. Nature.

[pgen-0030032-b002] Kong A, Gudbjartsson DF, Sainz J, Jonsdottir GM, Gudjonsson SA (2002). A high-resolution recombination map of the human genome. Nat Genet.

[pgen-0030032-b003] Matise TC, Sachidanandam R, Clark AG, Kruglyak L, Wijsman E (2003). A 3.9-centimorgan-resolution human single-nucleotide polymorphism linkage map and screening set. Am J Hum Genet.

[pgen-0030032-b004] d'Adda di Fagagna F, Reaper PM, Clay-Farrace L, Fiegler H, Carr P (2003). A DNA damage checkpoint response in telomere-initiated senescence. Nature.

[pgen-0030032-b005] Murnane JP, Sabatier L, Marder BA, Morgan WF (1994). Telomere dynamics in an immortal human cell line. EMBO J.

[pgen-0030032-b006] Henson JD, Neumann AA, Yeager TR, Reddel RR (2002). Alternative lengthening of telomeres in mammalian cells. Oncogene.

[pgen-0030032-b007] Londono-Vallejo JA, Der-Sarkissian H, Cazes L, Bacchetti S, Reddel RR (2004). Alternative lengthening of telomeres is characterized by high rates of telomeric exchange. Cancer Res.

[pgen-0030032-b008] Bailey SM, Goodwin EH, Cornforth MN (2004). Strand-specific fluorescence in situ hybridization: the CO-FISH family. Cytogenet Genome Res.

[pgen-0030032-b009] Cornforth MN, Eberle RL (2001). Termini of human chromosomes display elevated rates of mitotic recombination. Mutagenesis.

[pgen-0030032-b010] Hediger F, Berthiau AS, van Houwe G, Gilson E, Gasser SM (2006). Subtelomeric factors antagonize telomere anchoring and Tel1-independent telomere length regulation. EMBO J.

[pgen-0030032-b011] Therizols P, Fairhead C, Cabal GG, Genovesio A, Olivo-Marin JC (2006). Telomere tethering at the nuclear periphery is essential for efficient DNA double strand break repair in subtelomeric region. J Cell Biol.

[pgen-0030032-b012] Latt SA (1974). Localization of sister chromatid exchanges in human chromosomes. Science.

[pgen-0030032-b013] German J, Schonberg S, Louie E, Chaganti RS (1977). Bloom's syndrome. IV. Sister-chromatid exchanges in lymphocytes. Am J Hum Genet.

[pgen-0030032-b014] Opresko PL, von Kobbe C, Laine JP, Harrigan J, Hickson ID (2002). Telomere-binding protein TRF2 binds to and stimulates the Werner and Bloom syndrome helicases. J Biol Chem.

[pgen-0030032-b015] Bechter OE, Zou Y, Shay JW, Wright WE (2003). Homologous recombination in human telomerase-positive and ALT cells occurs with the same frequency. EMBO Rep.

[pgen-0030032-b016] Bechter OE, Zou Y, Walker W, Wright WE, Shay JW (2004). Telomeric recombination in mismatch repair deficient human colon cancer cells after telomerase inhibition. Cancer Res.

[pgen-0030032-b017] Dunham MA, Neumann AA, Fasching CL, Reddel RR (2000). Telomere maintenance by recombination in human cells. Nat Genet.

[pgen-0030032-b018] Mefford HC, Linardopoulou E, Coil D, van den Engh G, Trask BJ (2001). Comparative sequencing of a multicopy subtelomeric region containing olfactory receptor genes reveals multiple interactions between non-homologous chromosomes. Hum Mol Genet.

[pgen-0030032-b019] van Overveld PG, Lemmers RJ, Deidda G, Sandkuijl L, Padberg GW (2000). Interchromosomal repeat array interactions between chromosomes 4 and 10: A model for subtelomeric plasticity. Hum Mol Genet.

[pgen-0030032-b020] Lemmers RJ, Van Overveld PG, Sandkuijl LA, Vrieling H, Padberg GW (2004). Mechanism and timing of mitotic rearrangements in the subtelomeric D4Z4 repeat involved in facioscapulohumeral muscular dystrophy. Am J Hum Genet.

[pgen-0030032-b021] Trask BJ, Birren B, Green E, Hieter P, Myers R (1998). Fluorescence in situ hybridization. Genome analysis: A laboratory manual. Volume 4.

[pgen-0030032-b022] Lansdorp PM, Verwoerd NP, van de Rijke FM, Dragowska V, Little MT (1996). Heterogeneity in telomere length of human chromosomes. Hum Mol Genet.

