# Supplementary material for: Elevated Rates of Sister Chromatid Exchange at Chromosome Ends
Source: PLoS Genet. 2007 Feb 23;3(2):e32. doi: 10.1371/journal.pgen.0030032 (PMC1802831; doi:10.1371/journal.pgen.0030032)
Supplement: Figure S1 — (1.5 MB DOC) [file pgen.0030032.sg001.doc]

**SCE frequency**

20

11

9

8

X

7

6

5

2

**chromosome size (Mb)**

19

16

15

12

3

1

**Figure S1.** Correlation of chromosome size with SCE frequency. SCE frequency is equal to the total number of SCEs detected on each chromosome divided by the number of chromosomes counted. Approximate chromosome sizes (http://www.genome.ucsc.edu/) are plotted on the X-axis. Chromosome numbers are indicated adjacent to data points. Error bars were calculated as plus and minus the square root of the number of chromosomes counted.
