# Supplementary material for: Elevated Rates of Sister Chromatid Exchange at Chromosome Ends
Source: PLoS Genet. 2007 Feb 23;3(2):e32. doi: 10.1371/journal.pgen.0030032 (PMC1802831; doi:10.1371/journal.pgen.0030032)
Supplement: Table S1 — (81 KB DOC) [file pgen.0030032.st001.doc]

**Table S1.** CO-FISH data expressed per chromosome. The cell line analyzed and the probes are listed for each experiment. The number of CO-FISH configurations for each chromosome end is shown. Probes X and 7q only hybridize to one chromosome end, whereas probes Y and Z hybridize to multiple ends.

| normal cell line | probe | chromosome end | 0 SCE | 1 SCE in body | 1 SCE in terminus | 2 SCEs (1 in body, 1 in terminus) | total |
| --- | --- | --- | --- | --- | --- | --- | --- |
| GM08729 | X | 15q | 831 | 95 | 13 | 18 | 957 |
| GM08729 | Y | 1p | 118 | 39 | 1 | 8 |  |
|  |  | 3q | 170 | 45 | 2 | 6 |  |
|  |  | 5q | 156 | 39 | 3 | 2 |  |
|  |  | 6p | 5 | 0 | 0 | 0 |  |
|  |  | 6q | 135 | 41 | 4 | 3 |  |
|  |  | 8p | 60 | 9 | 1 | 3 |  |
|  |  | 11p | 68 | 10 | 0 | 2 |  |
|  |  | 12p | 7 | 3 | 0 | 0 |  |
|  |  | 15q | 174 | 15 | 2 | 6 |  |
|  |  | 16p | 4 | 0 | 0 | 1 |  |
|  |  | 19p | 19 | 2 | 1 | 0 |  |
|  |  | 20p | 46 | 4 | 2 | 1 |  |
|  |  | 20q | 7 | 0 | 0 | 2 |  |
|  |  | total | 969 | 207 | 16 | 34 | 1226 |
| GM08729 | Z | 3q | 95 | 22 | 1 | 2 |  |
|  |  | 6p | 82 | 16 | 2 | 2 |  |
|  |  | 6q | 7 | 2 | 0 | 0 |  |
|  |  | 9p | 105 | 11 | 0 | 1 |  |
|  |  | 11p | 6 | 0 | 1 | 0 |  |
|  |  | 12p | 64 | 7 | 1 | 0 |  |
|  |  | 15q | 103 | 10 | 0 | 1 |  |
|  |  | 16p | 58 | 7 | 0 | 2 |  |
|  |  | 19p | 52 | 2 | 0 | 1 |  |
|  |  | 20p | 147 | 7 | 0 | 1 |  |
|  |  | 20q | 100 | 6 | 0 | 4 |  |
|  |  | Xq | 9 | 0 | 1 | 0 |  |
|  |  | total | 828 | 90 | 6 | 14 | 938 |
| GM08729 | 7q | 7q | 793 | 153 | 7 | 13 | 966 |

|  |  |  |  |  |  |  |  |
| --- | --- | --- | --- | --- | --- | --- | --- |
| Bloom cell line | probe | chromosome end | even #s SCEs in body | odd #s SCEs in body | odd #s SCEs, 1 in terminus | even #s SCEs, 1 in terminus | total |
| GM16375 | Y | 1p | 28 | 27 | 0 | 1 |  |
|  |  | 3q | 83 | 78 | 4 | 2 |  |
|  |  | 5q | 46 | 34 | 1 | 2 |  |
|  |  | 6p | 69 | 68 | 4 | 2 |  |
|  |  | 6q | 55 | 60 | 3 | 4 |  |
|  |  | 8p | 7 | 7 | 0 | 0 |  |
|  |  | 11p | 74 | 60 | 0 | 2 |  |
|  |  | 12p | 6 | 8 | 0 | 0 |  |
|  |  | 15q | 52 | 67 | 3 | 1 |  |
|  |  | 19p | 28 | 15 | 2 | 1 |  |
|  |  | 20p | 6 | 1 | 0 | 1 |  |
|  |  | 20q | 4 | 0 | 0 | 1 |  |
|  |  | total | 458 | 425 | 17 | 17 | 917 |
| GM16375 | Z | 1p | 10 | 6 | 1 | 0 |  |
|  |  | 3q | 67 | 86 | 1 | 2 |  |
|  |  | 6p | 89 | 56 | 2 | 1 |  |
|  |  | 6q | 5 | 4 | 1 | 0 |  |
|  |  | 9p | 75 | 101 | 0 | 2 |  |
|  |  | 11p | 61 | 50 | 1 | 0 |  |
|  |  | 12p | 50 | 31 | 1 | 0 |  |
|  |  | 15q | 75 | 43 | 1 | 1 |  |
|  |  | 16p | 21 | 10 | 0 | 0 |  |
|  |  | 19p | 42 | 22 | 1 | 0 |  |
|  |  | 20p | 80 | 38 | 2 | 1 |  |
|  |  | 20q | 41 | 18 | 1 | 1 |  |
|  |  | Xq | 5 | 2 | 0 | 0 |  |
|  |  | total | 621 | 467 | 12 | 8 | 1108 |
