# Supplementary material for: Elevated Rates of Sister Chromatid Exchange at Chromosome Ends
Source: PLoS Genet. 2007 Feb 23;3(2):e32. doi: 10.1371/journal.pgen.0030032 (PMC1802831; doi:10.1371/journal.pgen.0030032)
Supplement: Table S2 — (24 KB DOC) [file pgen.0030032.st002.doc]

**Table S2.** SCE frequency corrected for double exchanges

| GM08729 |  | Observed | | | | Corrected | | | |
| --- | --- | --- | --- | --- | --- | --- | --- | --- | --- |
| probe | N | 0 SCE | 1 SCE in body | 1 SCE in terminus | 2 SCEs, 1 in body, 1 in terminus | 0 SCE | 1 SCE in body | 1 SCE in terminus | 2 SCEs, 1 in body, 1 in terminus |
| X- 10 Mb | 957 | 0.868 | 0.099 | 0.014 | 0.019 | 0.856 | 0.111 | 0.014 | 0.019 |
| Y- 110 kb | 1226 | 0.790 | 0.169 | 0.013 | 0.028 | 0.752 | 0.206 | 0.013 | 0.029 |
| Z- 10 kb | 938 | 0.883 | 0.096 | 0.006 | 0.015 | 0.872 | 0.107 | 0.006 | 0.015 |
| 7q- 50 kb | 966 | 0.821 | 0.158 | 0.007 | 0.013 | 0.789 | 0.190 | 0.007 | 0.014 |
