# Supplementary material for: Elevated Rates of Sister Chromatid Exchange at Chromosome Ends
Source: PLoS Genet. 2007 Feb 23;3(2):e32. doi: 10.1371/journal.pgen.0030032 (PMC1802831; doi:10.1371/journal.pgen.0030032)
Supplement: Text S1 — (64 KB DOC) [file pgen.0030032.sd001.doc]

**Supporting Information**

Hybridization efficiency

Chromosomes were only informative if they show three hybridization signals: one for the internal probe (X, Y, Z or 7q) and one telomere probe signal at each end. Our CO-FISH probes are relatively small: the internal probes hybridize to target sequences of 19-78 kb, while the telomere PNA hybridizes to telomere-repeat arrays estimated to range in length from ~2 kb to ~10 kb in normal human cells. Cot1 suppression of interspersed repeats (Alu, LINE) in the probes further reduces the size of the hybridization targets of the internal probes (see below). In every experiment, there was some degree of probe hybridization failure. This is common in FISH studies, and even more common when using small probes. We calculated the fraction of complete hybridization in each experiment by dividing the number of chromosomes scored by the expected number of chromosomes scored assuming no probe failure. These values are: Probe X: 0.69,

Probe Y: 0.19, Probe Z: 0.13, Probe 7q: 0.58. Probes X and 7q only are expected to hybridize to two chromosomes per cell, whereas the Y and Z probes have the potential to mark up to 14 and 15 chromosomes per cell, respectively.

Detection efficiency is expected to be less with the Y and Z probes than the X and 7q probes because of differences in target size (19, 25, 53, and 78 kb total target and 11, 19, 33, and 30 kb non-repetitive target, respectively). In addition, cells were only scored if at least one chromosome showed three requisite signals. Because probes Y and Z have the possibility of marking many more chromosomes per cell than do probes X and 7q, overall less efficiently hybridized cells are more often represented in the Y and Z experiments. For example, in the Y probe experiment, a cell with two out of fourteen hybridizing chromosomes would be scored, but would only represent a net hybridization efficiency of 0.14. In the X probe experiment, a cell with zero out of two signals would not be scored, and is thus absent from our hybridization efficiency calculations. Conversely, a cell with one signal would represent a net hybridization efficiency of 0.5. Thus, the difference in numbers of hybridizing chromosomes among the four experiments likely contributes to the overall hybridization-efficiency calculations.

Correction for missed double exchanges

Chromosomes with a cis telomere configuration experienced an odd number of SCEs, whereas those with a trans telomere configuration experienced an even number of SCEs. Although the majority of chromosomes we scored had no SCEs, some chromosomes experiencing two SCEs could be scored as zero SCE if both the SCEs occurred between the internal (green) probe and the telomeric probe on the opposite chromosome arm. To correct for undetected multiple SCEs, we applied the calculation derived by Cornforth and Eberle (2001) [1]:

μ = -ln(1-2γ)/2

where μ is the corrected frequency of SCEs and γ is the observed frequency of SCEs. The observed and corrected frequencies are listed in Table S2.

Corrected values were used to calculate the rate of SCE/bp per cell generation for each experiment in each interval. The SCE rate in the terminal interval is equal to the frequency of 1 SCE in the terminal interval plus the frequency of 2 SCEs (one terminal, one body) divided by the size of the interval. Similarly, the SCE rate in the body interval is equal to the frequency of 1 SCE in the body plus the frequency of 2 SCEs (one terminal, one body) divided by the size of the body. The body sizes vary among experiments since different chromosomes were labeled. Cells are grown in the presence of BrdU for one cell cycle only, so the rate is per cell generation.

For cell line GM08729,

Probe X: (0.014 + 0.019)/(10^7^ bp) = 3.3 X 10^-9^ SCE/(bp•cell generation)

Probe Y: (0.013 + 0.029)/(1.11 X 10^5^ bp) = 378 X 10^-9^ SCE/(bp•cell generation)

Probe Z: (0.006 + 0.015)/(10^4^ bp) = 2100 X 10^-9^ SCE/(bp•cell generation)

Probe 7q: (0.007 + 0.014)/(5 X 10^4^ bp) = 420 X 10^-9^ SCE/(bp•cell generation)

The above frequencies are cumulative; for example, the probe Y rate includes both the most telomeric interval demarcated by probe Z as well as the ~100 kb subtelomeric region. To calculate discrete rates of SCE in non-overlapping intervals, we subtracted the more terminal region from each overall measured region. Thus,

Probe Z (terminal 10 kb):

(0.006 + 0.015)/(10^4^ bp) = 2100 X 10^-9^ SCE/(bp•cell generation) (as above)

Probe Y (terminal 110 kb - terminal 10 kb = 100 kb):

[(0.013 + 0.029) - (0.006 + 0.015)]/(10^5^ bp) = 210 X 10^-9^ SCE/(bp•cell generation)

Probe X (terminal 10 Mb - terminal 110 kb = 9.9 Mb):

[(0.014 + 0.019) - (0.013 + 0.029)]/(9.9 X 10^6^ bp) ~ 0 SCE/(bp•cell generation)

In the Z-probe experiment, we also separately scored double telomeric probe signals (orange PNA probe signals on both chromatids at the same end), which could represent SCEs within the telomere proper (TSCE). Since these events could also arise due to incomplete degradation of the newly synthesized strand, we did not include them in our estimates of telomeric SCE rates. Doing so would have increased the rate of observed SCEs in the terminal ~10 kb by a factor of 1.2.

To calculate the rate of SCE in the body of the chromosome, we divided by the average size of the chromosomes scored for each experiment. We subtracted the analyzed terminal interval from total chromosome size to determine the size of the remainder of the chromosome body, and we subtracted the SCEs observed in the analyzed terminal region from the total observed. Thus, our estimation of the SCE rate in the body of the chromosome is conservative: the SCEs inferred to occur within the terminal interval of the other, unmarked chromosome end are not subtracted. Approximate chromosome sizes were determined by the March 2006 build of the human genome assembly (http://www.genome.ucsc.edu/).

Probe X body (chromosome 15) ~ 100 Mb - 10 Mb interval = 90 Mb

Probe Y body ~ 164.66 Mb - 0.11 Mb interval = 164.55 Mb

Probe Z body ~ 115.72 Mb - 0.01 Mb interval = 115.71 Mb

7q body ~ 159 Mb - 0.05 Mb interval = 158.95 Mb

Thus,

Body rate assayed with Probe X:

(0.111 + 0.019)/(9 X 10^7^ bp) = 1.4 X 10^-9^ SCE/(bp•cell generation)

Body rate assayed with Probe Y:

(0.206 + 0.029)/(1.6455 X 10^8^ bp) = 1.4 X 10^-9^ SCE/(bp•cell generation)

Body rate assayed with Probe Z:

(0.109 + 0.015)/(1.1571 X 10^8^ bp) = 1.1 X 10^-9^ SCE/(bp•cell generation)

Body rate assayed with 7q probe:

(0.19 + 0.014)/(1.5895 X 10^8^ bp) = 1.3 X 10^-9^ SCE/(bp•cell generation)

To calculate the average rate of SCE in the body of chromosomes, we adjusted for the number of chromosomes counted in each of the four experiments.

[((1.4 X 10^-9^ SCE/(bp•cell generation)) X 957 chromosomes) + ((1.4 X 10^-9^ SCE/(bp•cell generation)) X 1226 chromosomes) + ((1.1 X 10^-9^ SCE/(bp•cell generation)) X 938 chromosomes) + ((1.3 X 10^-9^ SCE/(bp•cell generation)) X 966 chromosomes)] / 4087 chromosomes = 1.3 X 10^-9^ SCE/(bp•cell generation)

Chromosome Twisting

Some chromosomes could be twisted at their centromeres when they are dropped onto a microscope slide. We were concerned that centromere twisting might influence our scoring of SCEs. For example, a twisted centromere would make a chromosome with trans-oriented telomeres appear in the cis-orientation, and vice versa. A chromosomal twist anywhere else along the chromosome would be visible, and not scored. To measure the amount of centromere twisting in our SCE assay, we took advantage of the consistent orientation of higher-order alpha satellite across centromeres. We designed a 25-mer oligonucleotide (5'-cttcgttggaaacgggatttcttcg-3') that hybridizes to the centromeres of chromosomes 1, 6, 12, and 16. We hybridized a biotin-labeled version of this oligonucleotide to the same preparation of GM08729 cells used in our subtelomeric SCE assays. Since alpha-satellite repeats are oriented head to tail across the centromere, an untwisted chromosome will have a single signal at the centromere and a twisted chromosome will have double centromere signals [2]. Three of 121 of chromosomes (2.4%) had a double centromere signal, suggesting centromere twisting. This small frequency of centromere twisting only affects the scoring of telomere signals relative to each other, i.e., the observed rate in the body of the chromosomes (see above), not the scoring of SCE between a subtelomeric probe and the telomere.

Body SCE calculation

Our values of SCE in the body of the chromosome are conservative; the above calculation includes events in the unmarked chromosome end. In the classic harlequin experiments, SCEs in the terminal regions would not be detected because they involve too little exchanged material to observe cytogenetically. Thus, the observed numbers of SCEs per cell per generation are expected to be lower in harlequin experiments. If we assume that the unmarked end has the same rate of SCE as the marked end, and subtract this number, our body frequency decreases:

X probe: {Body (95+18)-unmarked end (13+18)}/957= .086 SCEs/chromosome in body

Y probe: {Body (207+34)-unmarked end (16+34)}/1226= .16 SCEs/chromosome in body

Z probe: {Body (90+14)-unmarked end (6+14)}/938= .090 SCEs/chromosome in body

7q probe: {Body (153+13) -unmarked end (7+13)}/966= .15 SCEs/chromosome in body

Another difference between CO-FISH and harlequin chromosome experiments is the detection of chromosomes twisted at their centromeres. Chromatids twisted at their centromeres are visible in harlequin chromosomes due to the differential staining of chromatids; however, the CO-FISH methodology would miss any such twists. As reported above, we used an alpha satellite probe to estimate that twists occur at a rate of 0.024 per chromosome in our CO-FISH experiments. This phenomenon would not affect the rate of terminal SCEs, but does contribute to our counting of body SCEs. If we subtract 0.024 from each of the experiments, we get the following result:

X probe: 0.062 SCEs/chromosome in body

Y probe: 0.13 SCEs/chromosome in body

Z probe: 0.066 SCEs/chromosome in body

7q probe: 0.13 SCEs/chromosome in body

We must account for the average chromosome size in each experiment, as explained above, to determine the SCE/(bp•generation). Then to calculate the number of SCEs/cell, we multiply by 6.4 X 10^-9^ bp per human diploid cell.

X probe: (0.062 SCE/chromosome)/(9 X 10^7^ bp/chromosome) X (6.4 X 10^-9^ bp/cell) = 4.35 SCEs/(cell•generation)

Y probe: (0.13 SCE/chromosome)/(1.6455 X 10^8^ bp/chromosome) X (6.4 X 10^-9^ bp/cell) = 5.13 SCEs/(cell•generation)

Z probe: (0.066 SCE/chromosome)/(1.1571 X 10^8^ bp/chromosome) X (6.4 X 10^-9^ bp/cell) = 3.62 SCEs/(cell•generation)

7q probe: (0.13 SCE/chromosome)/(1.5895 X 10^8^ bp/chromosome) X (6.4 X 10^-9^ bp/cell) = 5.11 SCEs/(cell•generation)

Thus, our adjusted body SCE rates range from 3.6-5.1 SCEs/(cell•generation) among the four experiments. These are comparable to rates of SCEs/(cell•generation) in classic harlequin studies reported in the literature [3,4,5].

Excess of double SCEs

We noticed an apparent excess of chromosomes with double SCE configurations (Figure 2, configuration iv). We used a 2 X 2 contingency table to determine if our observed frequency of double SCEs deviated significantly from values expected if SCEs are independent events. Chi-square analysis showed that the frequency of double SCEs deviated significantly from expected values in each of four experiments with cell line GM08729 (p < 0.0001). 2 X 2 contingency tables using observed values corrected for multiple exchanges are shown below.

Probe X:

|  |  | body | |  |  |  |
| --- | --- | --- | --- | --- | --- | --- |
|  |  | 0 | 1 | total |  |  |
| terminus | 0 | 820 | 106 | 926 |  |  |
|  | 1 | 13 | 18 | 31 |  | **χ^2^ = 58.14** |
|  | total | 833 | 124 | 957 |  | p < 0.0001 |

Probe Y:

|  |  | body | |  |  |  |
| --- | --- | --- | --- | --- | --- | --- |
|  |  | 0 | 1 | total |  |  |
| terminus | 0 | 921 | 253 | 1174 |  |  |
|  | 1 | 16 | 36 | 52 |  | **χ^2^ = 65** |
|  | total | 937 | 289 | 1226 |  | p < 0.0001 |

Probe Z:

|  |  | body | |  |  |  |
| --- | --- | --- | --- | --- | --- | --- |
|  |  | 0 | 1 | total |  |  |
| terminus | 0 | 818 | 100 | 918 |  |  |
|  | 1 | 6 | 14 | 20 |  | **χ^2^ = 81.48** |
|  | total | 824 | 114 | 938 |  | p < 0.0001 |

Probe 7q:

|  |  | body | |  |  |  |
| --- | --- | --- | --- | --- | --- | --- |
|  |  | 0 | 1 | total |  |  |
| terminus | 0 | 762 | 184 | 946 |  |  |
|  | 1 | 7 | 13 | 20 |  | **χ^2^ = 25.51** |
|  | total | 769 | 197 | 966 |  | p < 0.0001 |

SCE is positively correlated with chromosome size

We measured CO-FISH configurations in over 4000 chromosomes in cell line GM08729. Chromosomes 1, 2, 3, 5, 6, 7, 8, 9, 11, 12, 15, 16, 19, 20 and the X chromosome had observed signals with probe X, Y, Z and/or 7q, and were therefore measured. We pooled per chromosome SCE data from all four experiments (Table S1) to determine the total SCE frequency. As shown in Figure S1, SCE total frequency is highly correlated with chromosome size.

References:

1. Cornforth MN, Eberle RL (2001) Termini of human chromosomes display elevated rates of mitotic recombination. Mutagenesis 16: 85-89.

2. Meyne J, Goodwin EH (1995) Direction of DNA sequences within chromatids determined using strand-specific FISH. Chromosome Res 3: 375-378.

3. Latt SA (1974) Localization of sister chromatid exchanges in human chromosomes. Science 185: 74-76.

4. Morgan WF, Crossen PE (1977) The frequency and distribution of sister chromatid exchanges in human chromosomes. Hum Genet 38: 271-278.

5. Londono-Vallejo JA, Der-Sarkissian H, Cazes L, Bacchetti S, Reddel RR (2004) Alternative lengthening of telomeres is characterized by high rates of telomeric exchange. Cancer Res 64: 2324-2327.
